# Chinese Bone Turnover Marker Study: Reference Ranges for C-Terminal Telopeptide of Type I Collagen and Procollagen I N-Terminal Peptide by Age and Gender

**DOI:** 10.1371/journal.pone.0103841

**Published:** 2014-08-12

**Authors:** Mei Li, Yan Li, Weimin Deng, Zhenlin Zhang, Zhongliang Deng, Yingying Hu, Weibo Xia, Ling Xu

**Affiliations:** 1 Department of Endocrinology, Key Laboratory of Endocrinology of Ministry of Health, Peking Union Medical College Hospital, Peking Union Medical College, Chinese Academy of Medical Sciences, Beijing, China; 2 Department of Laboratory, People's Hospital, Hubei Province, Wuhan, China; 3 Department of Geriatrics, General Hospital of Guangzhou Military Command, Guangzhou, China; 4 Department of Osteoporosis, Sixth People's Hospital, Shanghai Jiaotong University, Shanghai, China; 5 Department of Orthopedics, Second Affiliated Hospital of Chongqing Medical University, Chongqing, China; 6 Department of Obstetrics and Gynecology, Chinese Academy of Medical Sciences, Peking Union Medical College, Peking Union Medical College Hospital, Beijing, China; National University of Ireland, Galway (NUI Galway), Ireland

## Abstract

**Background:**

Bone formation marker procollagen I N-terminal peptide (PINP) and resorption marker C-terminal telopeptide of type I collagen (β-CTX) are useful biomarkers for differential diagnosis and therapeutic evaluation of osteoporosis, but reference values are required.

**Methods:**

The multi-center, cross-sectional Chinese Bone Turnover Marker Study included 3800 healthy volunteers in 5 Chinese cities. Serum PINP, β-CTX, parathyroid hormone (PTH) and 25OHD levels were measured by chemiluminescence assay. Lumbar spine and proximal femur BMD were measured by dual-energy X-ray absorptiometry. Serum PINP and β-CTX levels were assessed by age, gender, weight, recruitment latitude, levels of PTH and 25OHD.

**Results:**

Subjects (*n* = 1436, M∶F, 500∶936; mean age 50.6±19.6 years) exhibited non-normally distributed PINP and β-CTX peaking between 15–19 years, gradually declining throughout adulthood, elevating within 10 years of postmenopause, and then declining by age 70. In women between the age of 30 and menopause, median PINP and β-CTX levels were 40.42 (95% *CI*: 17.10–102.15) and 0.26 (95% *CI*: 0.08–0.72) ng/mL, respectively. β-CTX and PINP were positively linearly correlated (*r* = 0.599, *P*<0.001). β-CTX correlated positively (*r* = 0.054 and 0.093) and PINP correlated negatively (*r* = −0.012 and −0.053) with 25OHD and PTH (*P*<0.05).

**Conclusions:**

We established Chinese reference ranges for PINP and CTX. Chinese individuals exhibited high serum PINP and β-CTX levels between 15 and 19 years of age and at menopause, which gradually declined after 70 years of age.

## Introduction

Osteoporosis is characterized by decreased bone mineral density (BMD) and increased risk of bone fracture, affecting over 10% of men and nearly 20% of women in China [1]. In China and other developing countries, osteoporosis has reached epidemic proportions, with the occurrence of the disease increasing by nearly 300% over the last three decades [2]. The pathology of osteoporosis is diverse and linked with many risk factors for impaired bone remodeling; however, a critical risk factor for age-related osteoporosis is baseline BMD and age related bone loss, which has alarmingly been reported to be lower in Chinese-born individuals than those born in developed countries [3], [4]. Recently, biochemical bone turnover markers (BTM) have been employed to accurately and non-invasively assess decomposition and anabolism of bone tissues, thus providing an indication of osteoblast and osteoclast activities in bone remodeling critical to differential diagnosis of osteoporosis, assessment of bone fracture risk and effect evaluation of anti-osteoporosis therapy [5]. Reliable reference ranges for acceptable BTM, however, have not been developed for Chinese patients.

Bone remodeling is a dynamic process of spatiotemporal coupling that involves bone resorption mediated by osteoclasts and bone formation induced by osteoblasts [5]. Conventionally, osteoporosis diagnosis and treatment are assessed by BMD, measured by double-energy X-ray absorptiometry (DEXA) [6]. However, changes in BMD may not be immediately apparent after treatment, which need to take 12–24 months to indicate moderate efficacy [7]. As an alternative, serum BTM can be used to much more rapidly indicate the condition of bone loss or formation [7]. Furthermore, it has also been suggested that BTM may be useful in predicting bone fracture risk [8], potentially useful in osteoporosis and fracture prevention. Notably, Vasikaran *et al.* [7] highlighted in his review of BMD versus BTM utilities for osteoporosis management that the major limitation of wide employment of BTM was the lack of internationally agreed-upon standards for bone resorption and formation assessment, highlighting the need for precise and accurate standard reference ranges for normal BTM, as well as standards for osteoporosis and metabolic bone disease-associated BTM abnormalities.

Over the past two decades, numerous potential candidate markers of bone remodeling and their variability had been extensively examined [9]. Procollagen I N-terminal peptide (PINP) is a well-accepted marker of bone formation, produced by formation of type I collagen, a major component of bone matrix, by amino-terminal and carboxy-terminal splicing of type I procollagen in osteoblasts [10]. Conversely, C-terminal telopeptide of type I collagen (β-CTX) is considered as a marker of bone resorption, reflecting the degradation of type I collagen by osteoclasts to produce amino-terminal and carboxy-terminal fragments [11]. Recently, PINP and β-CTX have been recommended by the International Osteoporosis Foundation (IOF) and International Federation of Clinical Chemistry and Laboratory Medicine (IFCC) as the standard bone formation and resorption markers in the management of osteoporosis [12]. Furthermore, parathyroid hormone (PTH) and 25-hydroxyvitamin D (25OHD) have been suggested to be important hormones in the regulation of calcium and phosphorus metabolism, and thus play major roles in osteoporosis, though their relationship with BTMs remains unclear [10], [13]. Because PINP and β-CTX are key markers for differential diagnosis and therapeutic evaluation of osteoporosis, establishment of standard reference ranges for these BTMs is of great clinical importance.

This study was conducted in order to establish a set of comprehensive standard reference ranges for PINP and β-CTX levels in Chinese populations based on age and gender. Furthermore, the effect of location and latitude, body weight and circulating levels of PTH and 25OHD were assessed. These studies would provide a key preliminary step to establishment of internationally recognized standards for BTMs for use in Chinese osteoporosis prevention and treatment strategies.

## Subjects and Methods

### Study design

A total of 3800 age- and gender-stratified randomly selected healthy volunteers (capped at 100 subjects/strata) were recruited by the outpatient department of participating hospitals for a multi-center, parallel-group, cross-sectional study named the Chinese Bone Turnover Marker Study (CHBTM) between May 2011 and May 2012. Due to the higher prevalence of osteoporosis in females, females were recruited at a 2∶1 ratio to males. Subjects were recruited from hospitals in 5 cities located in different geographic regions of China (Beijing, Wuhan, Guangzhou, Shanghai, and Chongqing). Subjects were divided into subgroups according to age: 15–19 years, 20–29 years, 30–39 years, 40 years to menopause (40–49 years for men), menopause to 54 years (50–54 years for men), 55–59 years, 60–64 years, 65–69 years, 70–80 years, and >80 years. The Ethics Committees of all participating hospitals approved the study protocol (Peking Union Medical College Hospital, approval No. 2010-05-12; Hubei Province Hospital, approval No. 2010-05-18; General Hospital of Guangzhou Military Command, approval No. 2010-05-21; the Sixth People's Hospital of Shanghai Jiaotong University, approval No. 2010-05-22; the Second Affiliated Hospital of Chongqing Medical University, approval No. 2010-05-25). All participations were voluntary, and provided written informed consents. Written informed consents of children were obtained from guardians.

### Subjects

Subjects were included that (1) were aged ≥15 years; (2) were in good health according to medical history and current physical and laboratorial examinations; (3) exhibited body mass index (BMI) between 18 and 30 kg/m^2^; and (4) had normal anatomical structure of the lumbar vertebra suitable for DEXA assessment of BMD, with ≥three measurable vertebrae. Subjects were excluded that (1) had used bisphosphonates within the year directly preceding the study; (2) had used estrogen, strontium ranelate, raloxifene, parathyroid hormone preparations, or calcitonin in the three months directly preceding the study; (3) had undergone any therapy affecting bone metabolism for more than two weeks in the three months directly preceding the study, including therapy of androgen-stimulating, glucocorticoid or progestin; (4) had experienced bone fracture in the six months directly preceding the study; (5) had a history of metabolic bone disease other than postmenopausal bone loss, including hyperparathyroidism, hypoparathyroidism, Paget's disease and osteomalacia; (6) were pregnant or lactating in the two years directly preceding the study; (7) had abnormal thyroid function in the year directly preceding the study, identified as abnormal thyroid-stimulating hormone levels detected by super-sensitive thyroid function test; (8) had type 1 or type 2 diabetes; or (9) had severe liver and kidney damage, indicated by serum alanine aminotransferase more than two fold of the upper limit of normal values and serum creatinine level ≥150 µmol/L.

### Clinical assessments

For each subject clinical symptoms of osteoporosis, previous history of chronic disease, history of combined medication, menstrual, marital and child-bearing status, age at menopause, and family history were recorded. All subjects completed routine physical examination, including measurements of height and body weight.

### Blood sample collection

Fasting venous blood samples were collected from each subject between 8:00 and 9:00 AM local time and transferred to serum separation tubes (Covance, Indianapolis, IN, USA) supplied by the Department of Endocrinology, Peking Union Medical College Hospital (Beijing, China). Blood samples were placed at room temperature for 30 min to coagulate, and then centrifuged at 2500× g for 10 min to separate serum, which was stored at −70°C. Frozen serum was sent to the laboratory of the Department of Endocrinology, Peking Union Medical College Hospital (Beijing, China), and serum levels of PINP, β-CTX, PTH, and 25OHD were determined.

### Biochemical index determination

Unified detection of serum levels of β-CTX, PINP, PTH, and 25OHD was conducted in the laboratory of the Department of Endocrinology, Peking Union Medical College Hospital (Beijing, China) using a computer-controlled automatic analyzer (Roche cobas e 601) for chemiluminescence workstation with β-Crosslaps, total PINP, PTH and 25OHD with Elecsys reagent kits supplied by Roche Diagnostics (Basel, Switzerland). Detection of these parameters within each batch was completed simultaneously using the same reagent kits by the same technician, conducted according to the manufacturer-provided operating processes and special laboratory quality control procedures. Intra- and inter-assay coefficients of variation (CV) for detection of serum levels and minimum measureable values (MMV), respectively, were: PINP CVs = 1.2%–4.9% and 4.3%–6.5%, MMV = 5.0 ng/ml; β-CTX CV = 1.6%–3.0% and 1.3%–4.3%, MMV = 0.01 ng/ml; PTH CV = 1.5%–4.1% and 2.6%–6.2%, MMV = 1.2 pg/ml; 25OHD CV = 1.7%–7.8% and 2.2%–10.7%, MMV = 3.0 ng/ml. Liver and kidney functions, serum calcium, phosphorus, and alkaline phosphatase levels were detected using automated analyzers in each local hospital. Vitamin D deficiency, insufficiency and sufficiency were defined as serum 25OHD levels <20 ng/ml, ≥20 but <30 ng/ml, and ≥30 ng/ml, respectively [14].

### Bone density measurement

Bone density of the lumbar vertebra (postero-anterior projection, L1–4), left-sided proximal hip were measured using a Lunar Prodigy (GE Healthcare, Madison WI, USA) or Hologic QDR2000 (Hologic Inc., Bedford MA, USA) dual energy X-ray absorptiometry (DEXA) scanner. Bone density measurement, quality control, and data analyses were performed by a trained radiologist for each center blind to patient clinical data that analyzed each region of interest of bone density scanning. Bone density of the lumbar vertebra and proximal hip was automatically calculated by DEXA with an accuracy of 0.8–2.0%.

### Statistical analysis

Quantitative parameters were expressed as means ± standard deviation (SD), median, and 95% confidence interval, while qualitative variables were described as numbers and percentage values. All statistical analyses were performed using SPSS v.20 (SPSS Inc., Chicago, IL, USA). In order to investigate the characters of biomarkers of BTM, all parameters were described after age-, gender-, and site-based stratifications, and latitude of the sites of recruitment was in analyses. Distributions were described by scatter plot. Pearson's correlation analysis was used for normally distributed data, and Spearman's correlation analysis was used for non-normally distributed data, as well as multivariate analysis was performed, thus evaluating associations between BTMs with the age, body weight, height, recruitment location latitude, PTH and 25OHD levels. After base 10 logarithmic transformations, non-normally distributed data were fitted by linear regression. BTMs were compared among regions by Kruskal-Wallis test with Mann-Whitney *U* test for posthoc analysis. *P*-values less than 0.05 indicated statistical significance (*P*<0.05).

## Results

### Characteristics of the study population

**Figure 1:**
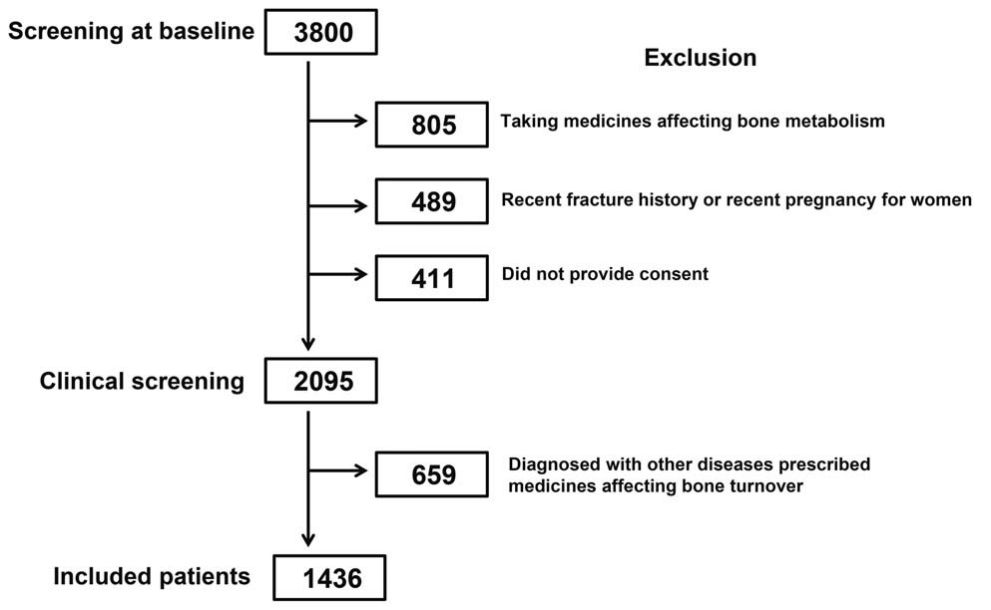
Subject screening and recruitment.

Of the total 3800 subjects screened, 805 (21.2%) were excluded for taking medications affecting bone densities, 489 (12.9%) were excluded for recent fracture or pregnancy, and 411 (10.8%) did not consent to participation. Of the remaining 2095 (55.1%) subjects, 659 (17.3%) were excluded due to medication use affecting bone turnover, and the remaining 1436 subjects were included in the study (Fig. 1). The study population included 936 women (65.2%) and 500 men (34.8%). We enrolled 189 (13.16%) participants from Beijing, 320 (22.28%) from Shanghai, 334 (23.26%) from Guangzhou, 217 (15.11%) from Chongqing, and 376 (26.18%) from Wuhan. The mean age of participants was 50.6±19.6 (range, 15–110) years, with mean body weight of 61.6±3.5 (range, 41.0–80.0) kg, and mean BMI of 23.1±5.3 (18.8–29.5) kg/m^2^. For the included postmenopausal women (*n* = 601), the mean age at menopause was 49.5±2.8 (age range of menopause, 40.0–56.0) years. Demographic and clinical characteristics for each age group were shown in Table 1.

**Table 1:** Characteristics and age distribution of the study population.

Characteristic	Female (*n* = 936)	Male (*n* = 500)	Total (*n* = 1436)
Age (years)	52.71±18.22(17–110)	46.45±21.33(15–105)	50.56±19.57(15–110)
Age group (years)			
15–19	17 (1.82%)	32 (6.40%)	49 (3.41%)
20–29	142 (15.17%)	122 (24.40%)	264 (18.38%)
30–39	88 (9.40%)	69 (13.80%)	157 (10.93%)
40–49	102 (10.9%)	54 (10.80%)	156 (10.86%)
50–54	92 (9.83%)	34 (6.80%)	126 (8.77%)
55–59	120 (12.82%)	35 (7.00%)	155 (10.79%)
60–64	123 (13.14)	33 (6.60%)	156 (10.86%)
65–69	92 (9.83%)	29 (5.80%)	121 (8.43%)
70–80	117 (12.5%)	58 (11.6%)	175 (12.19%)
>80	43 (4.59%)	34 (6.80%)	77 (5.36%)
Body weight (kg)	56.55±8.08 (41.0–84.0)	68.80±10.25 (43.0–98.5)	61.59±3.54 (41.0–80.0)
BMI (kg/m^2^)	22.96±3.30(18.90–29.50)	23.81±3.29 (18.8–29.03)	23.06±5.28 18.80–29.50)
BMD of L2–4 (g/cm^2^)	0.94±0.27 (0.40–1.98)	1.05±0.31 (0.66–1.64)	0.98±0.29 (0.4–1.98)
BMD of femoral neck (g/cm^2^)	0.81±0.40 (0.13–1.26)	0.95±0.65 (0.51–1.57)	0.86±0.51 (0.13–1.57)
Total hip BMD (g/cm^2^)	0.67±0.18 (0.20–1.86)	0.79±0.23 (0.27–1.59)	0.71±0.21 (0.20–1.86)

### Serum β-CTX, PINP, PTH, 25OHD levels and stratified analysis

PINP and β-CTX levels were non-normally distributed, with median levels of 49.36 (95% *CI*: 18.79–155.55) and 0.37 (95% *CI*: 0.11–0.90) ng/ml, respectively. Median PTH and 25OHD levels were 28.41 (95% *CI*: 8.62–71.08) and 18.39 (95% *CI*: 7.37–38.68) ng/ml, respectively. The mean serum 25OHD level was 17.9±8.1 ng/ml in the 936 female participants and 22.2±8.6 ng/ml in the 500 male participants. 68.6%, 19.2% and 12.2% of the female participants met the criteria for vitamin D deficiency, vitamin D insufficiency, and vitamin D sufficiency, respectively. 44.5%, 37.9% and 17.6% of the male participants met the criteria for vitamin D deficiency, vitamin D insufficiency, and vitamin D sufficiency, respectively.

**Figure 2:**
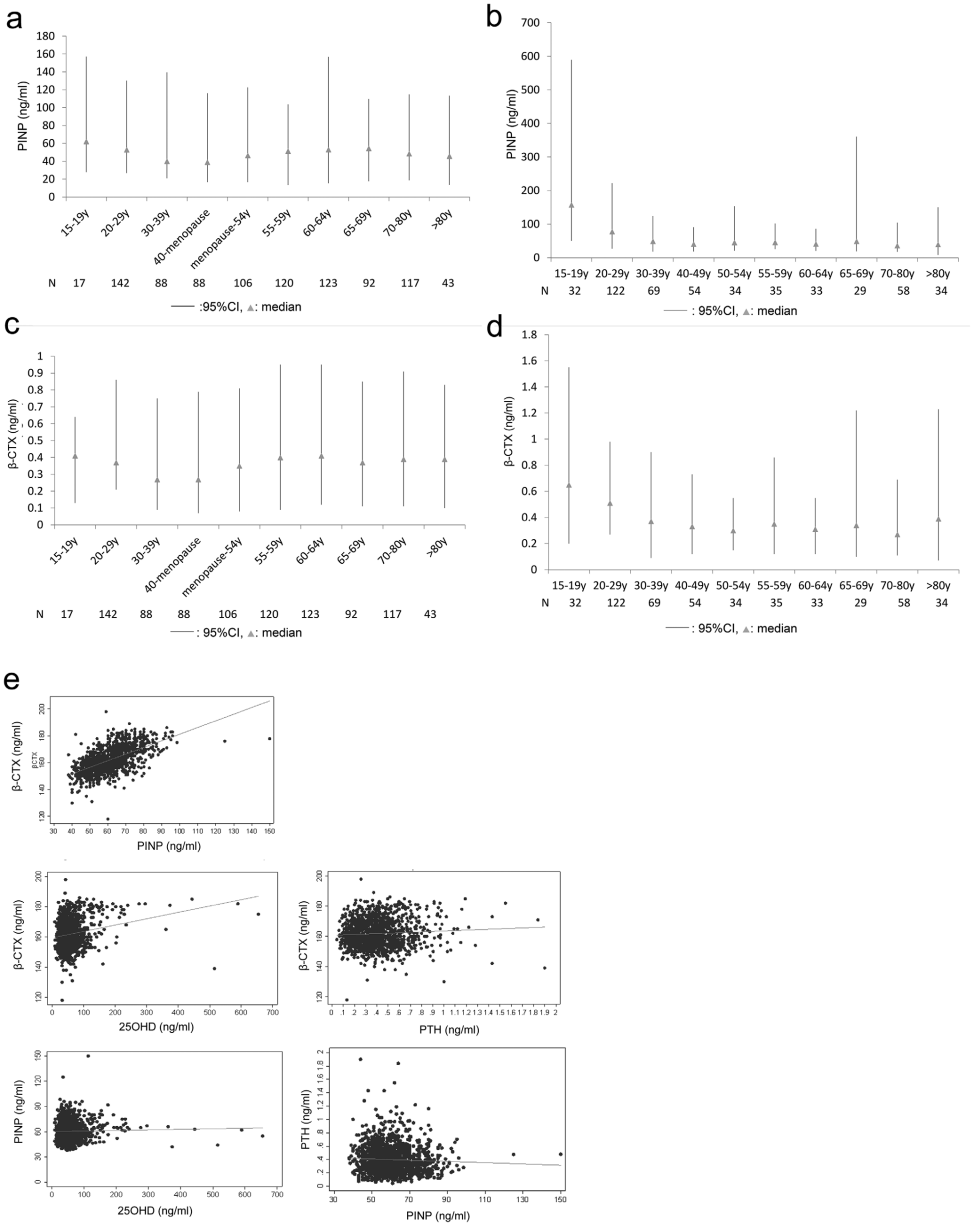
PINP and β-CTX levels in different groups and the results of correlation analysis. a PINP levels in female age groups. b PINP levels in male age groups. c β-CTX levels in female age groups. d β-CTX levels in male age groups. e The correlation between β-CTX, PINP, PTH and 25OHD levels in participants.

Age-stratified analysis revealed relatively high levels of PINP and β-CTX in women between 15 and 19 years of age, thereafter gradually declining with age. Significantly elevated levels of PINP and β-CTX were observed again in women from menopause to 60 years of age; however, PINP and β-CTX levels slowly declined in women beyond ages of 70 and 65 years, respectively (Fig. 2A and 2C). Similarly, PINP and β-CTX peaked in men between 15 and 19 years of age, thereafter, levels of both markers gradually declined. PINP and β-CTX remained at low levels in men from 40 to 69 years of age, declining further after age 70 (Fig. 2B and 2D). PINP and β-CTX levels in men between the age of 15 and 19 years were significantly higher than in women in the same age bracket (*P*<0.001, Fig. 2A and 2C). PINP and β-CTX levels in postmenopausal women aged <64 years were significantly higher than those in men aged 50–64 years (*P*<0.001, Fig. 2B and 2D).

### Correlations among β-CTX, PINP, PTH, and 25OHD

Spearman's correlation analysis revealed a significant positive correlation between PINP and β-CTX levels in the whole study population (*r* = 0.599, *P*<0.001) (Fig. 2E). After base 10 logarithmic transformations, a simple linear equation (*y* = 1.869+0.191*x*, *R* ^2^ = 0.362) was fitted, indicating the close coupling relationship between bone resorption and bone formation. PINP levels negatively correlated with 25OHD and PTH levels, with low correlation coefficients (*r* = −0.012 and −0.053, both *P*<0.05) (Fig. 2E). β-CTX positively correlated with PTH and 25OHD levels, though correlation coefficients were relatively low (*r* = 0.054 and 0.093, *P*<0.05 and 0.001) (Fig. 2E).

### Correlations of β-CTX and PINP levels with recruitment location latitude and body weight

No significant differences were observed in PINP and β-CTX levels in either men or women among the 5 cities with different latitudes (all *P*>0.05) after the age, body weight and height were adjusted (Table 2). PINP and β-CTX levels did, however, positively correlated with body weight, exhibiting low correlation coefficients (*r* = 0.097 and 0.19, both *P*<0.001).

**Table 2:** PINP and β-CTX levels in male and female subjects by sites of recruitment latitude.

City (Latitude)	PINP (ng/ml)		β-CTX (ng/ml)	
	Female	Male	Female	Male
Beijing	43.01(15.27,96.96)	40.40(18.23,94.15)	0.28(0.09,0.79)	0.28(0.10,0.68)
(39°54′27″N)	(*n* = 93)	(*n* = 96)	(*n* = 93)	(*n* = 96)
Shanghai	53.08(14.92,135.43)	29.74(17.31,56.74)	0.41(0.11,0.88)	0.25(0.10,0.39)
(31°13′56″N)	(*n* = 307)	(*n* = 13)	(*n* = 307)	(*n* = 13)
Wuhan	46.02(20.95,105.18)	47.85(24.10,163.43)	0.35(0.12,0.75)	0.39(0.17,1.01)
(29°58′20″N)	(*n* = 207)	(*n* = 169)	(*n* = 207)	(*n* = 169)
Chongqing	53.07(20.66,120.34)	47.07(20.08,125.11)	0.42(0.12,0.91)	0.39(0.12,1.21)
(29°36′21″N)	(*n* = 134)	(*n* = 83)	(*n* = 134)	(*n* = 83)
Guangzhou	48.33(19.77,116.69)	76.46(29.41,402.45)	0.33(0.08,0.82)	0.45(0.15,1.14)
(23°06′32″N)	(*n* = 195)	(*n* = 139)	(*n* = 195)	(*n* = 139)

There were no significant differences in the β-CTX and PINP concentrations among different cities after the age, body weight and height were adjusted.

## Discussion

As the first comprehensive, multi-center, age- and gender-stratified report of standard reference ranges for BTMs PINP and β-CTX in five geographically distant Chinese cities, this study is uniquely capable of describing the BTM characteristics of the Chinese populace. This study provides novel indications of high levels of bone turnover in both men and women during late adolescence and in women during menopause. Bone turnover levels also gradually declined in both men and women throughout adulthood and, more dramatically, above 70 years of age. As the age range in our study was very wide (15–110 years old), we were able to study BTMs in an elderly population. Furthermore, PINP and β-CTX levels correlated with each other and with body weight. Additionally, β-CTX level correlated positively (*r* = 0.054 and 0.093, *P*<0.05 and 0.001) and PINP level correlated negatively (*r* = −0.012 and −0.053, both *P*<0.05) with serum 25OHD and PTH levels. However, the correlation coefficients between β-CTX, PINP and 25OHD, PTH levels were extremely low (r<0.1), so the clinical significance of this correlations was limited. This study provides reference interval values for PINP and CTX in healthy Chinese adults, which will contribute to appropriate assessment of bone turnover and suggest appropriate treatment targets.

Significant overlap had been reported between PINP and β-CTX levels in both healthy individuals and subjects with osteoporosis, suggesting that BTMs such as PINP and β-CTX had variant utilities for diagnosis of osteoporosis, prediction of bone turnover, and prevention of fracture, though these relationships were not fully understood [15], [16]. As a result, primary diagnosis of osteoporosis may not be the most notable use of BTMs. Notably, elevation of PINP and β-CTX did, however, reliably predict the presence of bone diseases associated with malignant tumors, including breast cancer, prostate cancer, and lung cancer and early signs of metabolic bone diseases [17]–[20]. Therefore, standard reference ranges for PINP and β-CTX would make these markers much more useful for differential diagnosis of osteoporosis. Furthermore, establishment of standard reference ranges for PINP and β-CTX would facilitate the development of improved treatment strategies for osteoporosis by allowing dynamic assessment of changes in bone metabolism in the immediate period following treatment [21]–[23]. However, reference ranges for PINP and β-CTX may be different between age groups, duration of disease, and physiological status. As the present study was a cross-sectional one, it did not allow dynamic assessment of changes in bone metabolism during biological variation. Therefore, more prospective studies are needed to clarify these issues.

As in North American and European populations [24]–[26], in this study BTM levels changed predictably with age and gender in the Chinese population. The highest rate of bone turnover in both genders occurred in late adolescence and thereafter declined, consistent with a previous study of 1541 Chinese youths by Guo *et al.* [27] indicating that Chinese adolescents aged 16 to 19 exhibited the greatest BMD changes, most notably in males. Furthermore, significantly higher levels of PINP and β-CTX were detected in postmenopausal women (between menopause and 64 years of age) than in men between 50 and 64 years of age, consistent with previous reports indicating that significant bone density loss was associated with a rapid reduction in estrogen of menopausal women [28]. Notably, bone turnover level gradually reduced in both men and women aged over 70 years, potentially associated with the dysfunction of osteoblasts and osteoclasts and altered calcium metabolism in the oldest-old [29]. Thus, the current results were generally consistent with those found in other populations and previous reports of Chinese patients.

Notably, we observed a significant positive and linear correlation between PINP and β-CTX levels and between these BTMs and body weight, suggesting that bone turnover closely reflected coupling processes of bone resorption and bone formation. In a study of 87 healthy Brazilian males aged 10–18 years, similar positive correlations were observed between body weight and serum carboxyterminal telopeptide (*r* = 0.40) and osteocalcin levels (*r* = 0.21) that may have resulted from adipokine effects on the skeletal bone targets, altering sympathetic impulses and exerting paracrine effects bone tissues [30]. Correlations between BTM levels and geographic locations were poor in the present study, which remained controversial, perhaps due to nutritional and lifestyle variations more than the nature of the location itself [4]. In a study of 637 healthy premenopausal women from the United Kingdom, France, Belgium, and the United States, β-CTX was significantly higher in France relative to the United Kingdom, and PINP was higher in France and Belgium relative to the United Kingdom, with researchers similarly concluded that these variations were predominantly due to lifestyle rather than genetic or geographic variations [31]. This was further confirmed by the limited variation exhibited among 5 European centers in a report by Blumsohn *et al.* [32], and higher bone turnover levels in North America than those in German and Spanish women [33]. Thus, further work would be required to determine the precise lifestyle and nutritional factors responsible for raised osteoporosis risk; however, implementation of BTM measurements would be certain to improve the feasibility of such research.

BTMs ranges in healthy premenopausal women had garnered increasing recent research attention, potentially due to therapeutic goals of restoring normal BTM levels in patients with osteoporosis and metabolic bone diseases [34]. Interestingly, the median levels of PINP and β-CTX in the 176 premenopausal women from 30 years old to menopause in the current study (40.42 and 0.26 ng/mL, respectively) were very similar to those reported in 194 premenopausal European women aged 35–39 years reported by Eastell *et al.* in 2012 [9], 153 women aged 35–45 years reported by Glover *et al.* in 2008 [35], 637 premenopausal women aged 35–39 years reported by Glover *et al.* in 2009 [31], and in premenopausal women aged 28–45 years reported by de Papp *et al.* in 2007 [36], suggesting that the range of PINP and β-CTX levels in premenopausal women, as the normal reference range, had no apparent racial variation. We studied a wide range of Chinese women from five cities, and found that the BTM characteristics of these participants were not very different from the results reported by similar studies of European and American women in studies using similar techniques [9], [30], [34], [35]. However, we cannot exclude the possibility that these studies enrolled non equivalent populations based on inclusion and exclusion criteria different to that we employed in participant screening.

The current study, however, was limited by the failure to record dietary calcium intake and daily exercise, thus we could not assess the impact of lifestyle on BTMs. Furthermore, the smaller numbers of participants in certain subgroups (e.g., 17 women and 32 men aged 15–19; 13 men from Shanghai) had limited the significance and applicability of these analyses. We also did not record serum estrogen and androgen levels, and bone density was measured using Lunar or Hologic dual-energy X-ray bone absorptiometry scanners, which may exhibit some variation due to equipment types or calibration. Additionally, all study subjects were urban residents, which avoided the effect of variations of living environments and habits on BTMs levels but may limited broad applicability to rural populations due to lifestyle and nutritional variations, meriting further investigation.

## Conclusions

This large-scale, multi-center, cross-sectional study provided a novel, comprehensive determination of standard reference ranges for the bone formation marker PINP and bone resorption marker β-CTX in Chinese men and women by age. High bone turnover levels were detected in both men and women during late adolescence and in women at menopause, and bone turnover gradually declined in both men and women after age 70.
